# Trends in 5-year community management of persons with dementia in Korea, 2003–2016

**DOI:** 10.1371/journal.pone.0342459

**Published:** 2026-03-11

**Authors:** Wonjae Sung, Hyuk Sung Kwon, Jeewon Suh, Im-Seok Koh, Keun U. Park, Hojin Choi

**Affiliations:** 1 Department of Neurology, Hanyang University College of Medicine, Seoul, Republic of Korea; 2 Department of Neurology, Hanyang University Guri Hospital, Hanyang University College of Medicine, Guri, Republic of Korea; 3 Department of Neurology, Dongguk University Ilsan Hospital, Goyang, Korea; 4 Department of Neurology, National Medical Center, Seoul, Republic of Korea; 5 Claim Data Analyst, Seoul, Republic of Korea; Taipei Veterans General Hospital, TAIWAN

## Abstract

**Background:**

The community 5-year management rate, defined as the proportion of patients with dementia who remain in community-based informal care without long-term institutionalization 5 years after diagnosis reflects the effectiveness of national dementia strategies and social care systems.

**Objective:**

To examine national trends in the 5-year community management rate of dementia and assess whether disparities in dementia care outcomes have changed by demographic, socioeconomic, and clinical characteristics.

**Methods:**

This **r**etrospective, population-based cohort study used a customized research database from the Korean National Health Insurance Service (2003–2021). Subgroup analyses were performed by age, sex, income, region (metropolitan vs. non-metropolitan), Charlson Comorbidity Index, diagnosing department (neurology/psychiatry vs other). The study population included patients newly diagnosed with dementia per annum during the study period. The primary outcome was the proportion of patients remaining in community 5 five years after diagnosis, without long-term institutionalization. Secondary outcomes included disparities in management rates across subgroups.

**Results:**

Overall 779,558 patients were included. The 5-year community management rate showed continued improvement over time. Disparities by sex, residence, and income narrowed steadily between 2003 and 2016. Patients diagnosed in neurology or psychiatry consistently had higher management rates than those diagnosed in other departments, and this gap widened over time.

**Conclusions:**

Community management rates are influenced by social and personal factors. While disparities by sex, income, and residence decreased, persistent differences by comorbidity and diagnosing department highlight the need for targeted policy interventions. The 5-year community management rate may serve as a meaningful indicator of real-world dementia care outcomes.

## Introduction

As the global prevalence of dementia continues to rise, the associated socioeconomic burden is also projected to increase substantially.[1] In response, many countries have developed National Dementia Plans (NDPs) to improve quality of life for individuals with dementia and their caregivers while mitigating the societal and economic impact of this disease.[2] These plans aim to address the rapidly escalating costs of dementia care through coordinated policy interventions and enhanced service delivery.[3]

In South Korea, national-level efforts to address the growing burden of dementia began as early as 2008 with the implementation of the first NDP, which has since evolved in both scope and structure.[4] The government further reinforced this commitment in 2017 by announcing the “National Responsibility Policy for Dementia Care” initiative, pledging to take primary responsibility for supporting individuals affected by dementia and their families.[5] Currently, Korea is implementing its Fourth NDP (2021–2025) under the vision of “Completing and Realizing the National Responsibility for Dementia.” [6] This plan builds on previous initiatives and emphasizes comprehensive support across prevention, early detection, treatment, long-term care, and social integration.[6]

Dementia management can be evaluated through neuropsychological testing, neuroimaging, and laboratory examinations at the individual level. However, applying such resource-intensive assessments to large populations is often impractical due to financial and logistical constraints. While monitoring the incidence and prevalence offers insights into disease trends, these indicators are limited in capturing the quality of care or lived experiences of people with dementia. To address this gap, we previously proposed a novel monitoring indicator—the “in-community management rate”—defined as the proportion of patients with dementia who continue to receive informal care within their communities without being institutionalized in nursing facilities or geriatric hospitals for >3 consecutive months.[7] This metric provides a population-level proxy for evaluating sustained community-based care, a key objective of national dementia strategies.

In our previous study, we examined national trends in dementia care by analyzing the 5-year in-community management rate across various demographic and clinical subgroups, corresponding with the progression of Korea’s NDPs.[7] We demonstrated a gradual increase in both the number of newly diagnosed dementia cases and proportion of patients managed within the community following the initial implementation of the NDP. While disparities in in-community management by sex and residential region showed signs of narrowing, differences related to the comorbidity burden and income level tended to widen over time.[7]

To further expand on these findings, the present study extends the observation period by an additional 3 years to evaluate whether these subgroup disparities persist over the longer term. Furthermore, this investigation demonstrates the impact of the large-scale National Responsibility Policy for Dementia Care launched in 2017 by providing a quantitative indicator of community management outcomes. In addition, we examined whether in-community management outcomes differed depending on the department of initial diagnosis by comparing dementia specialists (neurology or psychiatry) with non-specialist departments.

## Materials and methods

### Data source

This study was conducted using de-identified customized research data extracted from the National Health Insurance Database between January 1, 2003, and December 31, 2021 (NHIS-2022-1-665). This database is based primarily on the Korean National Health Insurance Service (NHIS), a single government insurer that covers 97% of the Korean population; claims from the remaining 3% (covered by the Medical Assistance Program or Medial Care for Patriots and Veterans Affairs Scheme) are reviewed by the NHIS.[7] The customized database is representative of the transmission data provided by de-identifying health and long-term care insurance data. The database provides healthcare utilization information for both inpatients and outpatients and includes patient demographics, diagnoses, diagnostic procedures, and prescribed medications. The Korean Classification of Disease (KCD), 5–7^th^ editions, and a modification of the International Classification of Disease and Related Health Problems, 10^th^ revision, were used to code the diagnoses. Data on demographics (including age, sex, income, and residence), accompanying diagnostic codes including the Charlson Comorbidity Index (CCI), and hospitalization records including the diagnosis department were collected using the NHIS coding system.

### Study population

We accessed the NHIS datasets on December 7, 2022, and identified patients who were first diagnosed with dementia between 2003 and 2016 using KCD-5, KCD-6, or KCD-7 codes from the claims data. Dementia-related diagnostic codes were registered in the health insurance database when a patient with suspected dementia visited a hospital. This diagnostic code remained in the database when patients visited again to check the test results, including neuropsychological assessments, laboratory tests, and neuroimaging. Therefore, to reduce false positives when selecting patients with dementia, they were defined as those with a history of three or more outpatient visits or admissions with a dementia-related diagnostic code. The first recorded visit for these patients was the one wherein dementia was diagnosed. Dementia-related diagnostic codes were F00 (Dementia in Alzheimer disease), F01 (Vascular dementia), F02 (Dementia in other diseases classified elsewhere), F03 (Unspecified dementia), G30 (Alzheimer disease), G31.00 (Behavioral variant frontotemporal dementia), G31.01 (Semantic variant primary progressive aphasia), G31.02 (Nonfluent primary progressive aphasia), G31.03 (Logopenic primary progressive aphasia), G31.04 (Primary progressive aphasia), and G31.82 (Dementia with Lewy bodies). Participants with any claims record of a dementia-related diagnostic code in 2002 were excluded as part of the washout period.

### Community management rate

We defined “community management” as informal care provided by patients or caregivers following a dementia diagnosis. The duration of community management spans from the dementia diagnosis to its end, which occurs when patients die or are admitted to a nursing home or medical facility for >3 months. The community management rate is the proportion of patients with dementia receiving community management compared to that of the total number of patients with dementia in the population.

We followed the community management probability for patients diagnosed with dementia between 2003 and 2007 (Supplementary figure 1). There was no significant difference after 15 years. However, it is difficult to use 15 years as the standard follow-up period because of its length. We analyzed changes in the community management rate at 5-year intervals.

### Ethical approval

This study was approved by the Institutional Review Board of Hanyang University Guri Hospital (2022–04–039). All personal information in the NHIS database was de-identified, and the requirement for informed consent was waived.

### CCI

We used the 10^th^ revision of the International Classification of Diseases (ICD-10) version of the CCI, which includes 17 diagnostic categories: acute myocardial infarction, congestive heart failure, peripheral vascular disease, cerebrovascular accident, dementia, pulmonary disease, connective tissue disorder, peptic ulcer, liver disease, diabetes, diabetes complications, paraplegia, renal disease, cancer, metastatic cancer, severe liver disease, and human immunodeficiency virus infection. In this study, as all participants had dementia, the other 16 diagnostic categories were weighted and calculated as CCI scores. The weighted values and corresponding ICD-10 codes are listed in Supplementary Table 1.[8]

### Statistical analysis

Community 5-year management rate was calculated for patients diagnosed with dementia between 2003 and 2013. Descriptive statistics of the study population are presented. All participants were divided according to age (five groups: < 50, 50–59, 60–69, 70–79, and ≥ 80), sex, CCI (three groups: 0, 1–2, and ≥ 3), residence (metropolitan vs non-metropolitan), income (quintiles), diagnosing department (neurology or psychiatry vs others) for comparison. Continuous variables are expressed as means ± standard deviation, and categorical variables are expressed as percentages or frequencies. The community management rate was compared using the chi-square test 5 years following dementia diagnosis with Bonferroni correction. We applied interrupted time-series analysis (ITS) to evaluate changes in the annual 5-year community management rate associated with major national dementia policy implementations, specifying two intervention knots in 2008 and 2012. All statistical analyses were performed using SAS version 9.4 (SAS Institute Inc., Cary, NC, USA), and Python 3.9.11, with the SciPy library, and P < 0.05 was considered statistically significant.

## Results

**Fig 1:**
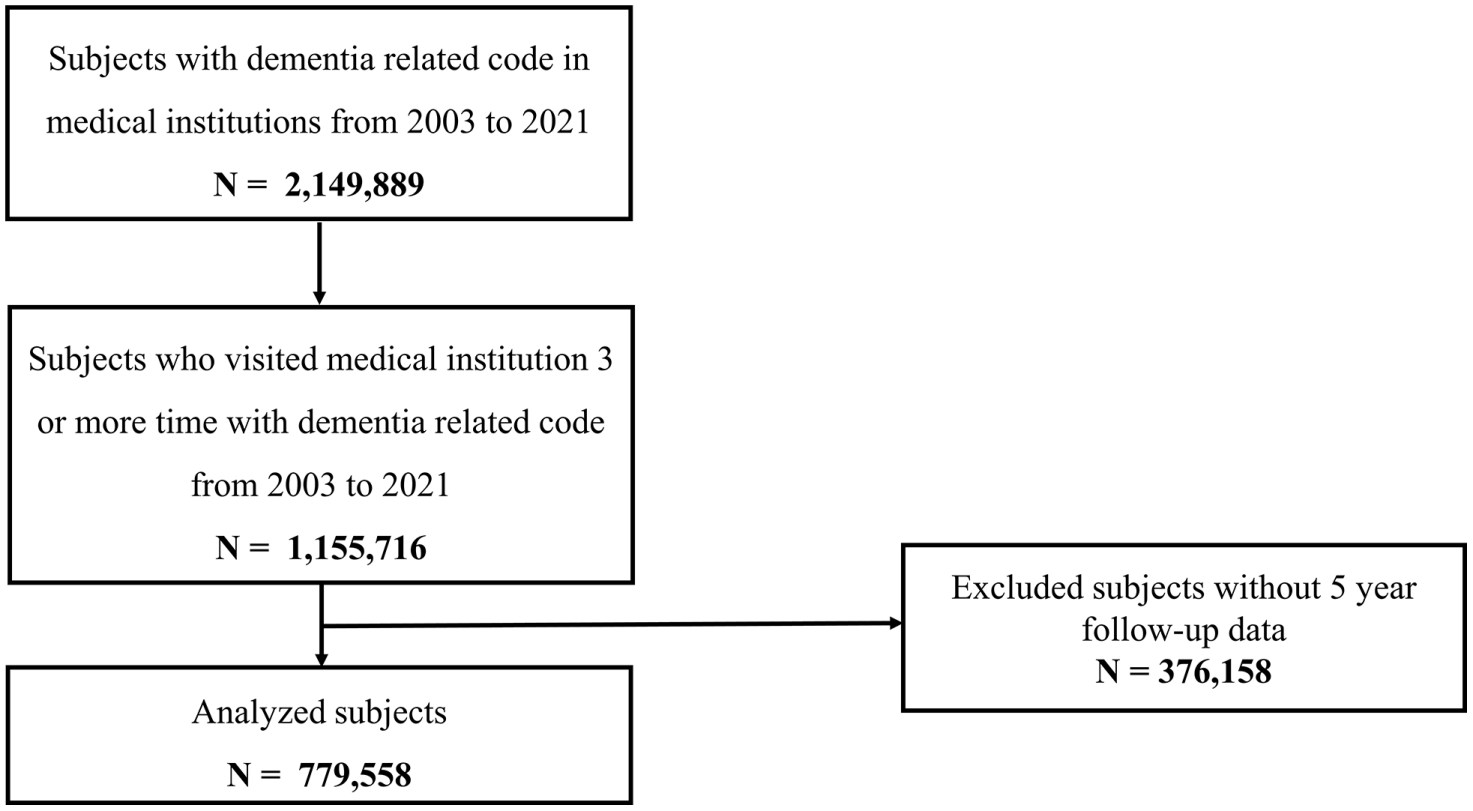
Flowchart of analyzed dementia patients from the National Health Insurance Database.

Dementia-related diagnostic codes were documented for 2,149,889 individuals who sought medical care between 2003 and 2021. Among them, 1,155,716 had at least three medical encounters associated with dementia-related diagnostic codes. The final study cohort, comprising 779,558 individuals diagnosed between 2003 and 2016, of whom 64.4% were female, was selected for analysis of the 5-year in-community management rate (Fig 1).

**Fig 2:**
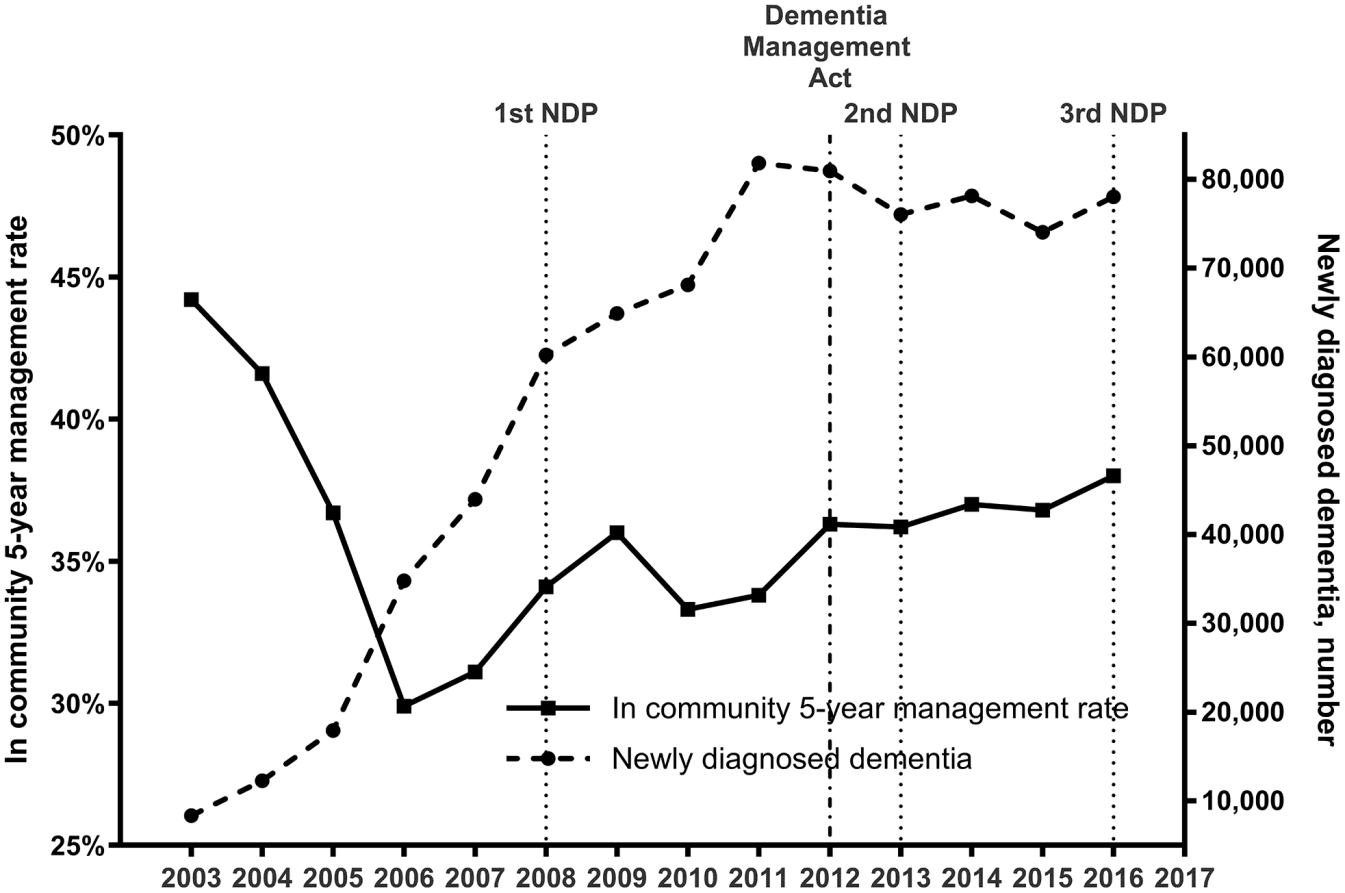
Five-year community management rate and number newly diagnosed patients with dementia from 2003 to 2016.

Table 1 summarizes the demographic characteristics and comorbidity profiles of the study population. From 2014 to 2016, the number of patients newly diagnosed with dementia remained at a level similar to that observed in 2013 (Fig 2). Although the crude 5-year community management rate declined slightly in 2015, it has exhibited a modest upward trend in the upper 30% range from 2012 onward. The age-standardized 5-year community management rate followed a similar trajectory (Supplementary figure 2). The ITS analysis, which evaluates the impact of national dementia policies, revealed significant upward shifts in the community 5-year management rate following both interventions (Supplementary figure 3), suggesting an immediate positive response to policy implementation.

**Table 1:** Demographic characteristics and comorbidities of subjects diagnosed with dementia in each year.

	Overall (n = 779, 558)	2003 (n = 8, 333)	2004 (n = 12, 269)	2005 (n = 17, 918)	2006 (n = 34, 783)	2007 (n = 43, 947)	2008 (n = 60, 229)	2009 (n = 64, 888)	2010 (n = 68, 116)	2011 (n = 81, 831)	2012 (n = 80, 938)	2013 (n = 76, 045)	2014 (n = 78, 139)	2015 (n = 74, 045)	2016 (n = 78, 034)
Demographics															
Age, years	75.6 ± 10.4	72.6 ± 10.3	73.4 ± 9.8	74.6 ± 9.7	75.0 ± 10.1	75.3 ± 10.2	75.2 ± 10.3	75.1 ± 10.3	75.7 ± 10.2	75.9 ± 10.3	75.8 ± 10.5	75.7 ± 10.5	75.86 ± 10.5	76.14 ± 10.5	76.26 ± 10.6
Sex, female	498,549 (64.0)	5,059 (60.7)	7,488 (61.0)	11,216 (62.6)	22,898 (65.8)	28,760 (65.4)	39,372 (65.4)	41,647 (64.2)	44,039 (64.7)	53,210 (65.0)	51,779 (64.0)	48,445 (63.7)	49,206 (63.0)	46,517 (62.8)	48,883 (62.6)
Income, quintile															
<40%	181,240 (23.2)	2,028 (24.3)	3,051 (24.9)	4,308 (24.0)	6,132 (17.6)	8,564 (19.5)	12,296 (20.4)	13,667 (21.1)	16,330 (24.0)	20,230 (24.7)	20,491 (25.3)	18,834 (24.8)	18,502 (23.7)	17,987 (24.3)	18,820 (24.1)
40–60%	87,790 (11.3)	1,032 (12.4)	1,491 (12.2)	2,558 (14.3)	3,003 (8.6)	4,614 (10.5)	6,463 (10.7)	7,354 (11.3)	7,662 (11.2)	9,000 (11.0)	9,016 (11.1)	8,596 (11.3)	9,151 (11.7)	8,693 (11.7)	9,157 (11.7)
>60%	373,764 (47.9)	5,173 (62.1)	7,562 (61.6)	10,453 (58.3)	12,944 (37.2)	20,077 (45.7)	27,595 (45.8)	30,169 (46.5)	31,011 (45.5)	38,217 (46.7)	38,740 (47.9)	36,666 (48.2)	39,179 (50.1)	37,064 (50.1)	38,914 (49.9)
Others*	136,764 (17.5)	101 (1.2)	166 (1.4)	600 (3.3)	12,710 (36.5)	10,696 (24.3)	13,881 (23.0)	13,704 (21.1)	13,116 (19.3)	14,388 (17.6)	12,698 (15.7)	11,953 (15.7)	11,307 (14.5)	10,301 (13.9)	11,143 (14.3)
Residence															
Metropolitan	205,329 (37.4)	3,195 (38.3)	4,473 (36.5)	6,495 (36.2)	10,796 (31.0)	14,759 (33.6)	22,068 (36.6)	24,033 (37.0)	25,608 (37.6)	32,053 (39.2)	32,054 (39.6)	29,795 (39.2)	30,750 (39.4)	29,608 (40.0)	32,100 (41.1)
Non-metropolitan	343,968 (62.6)	5,138 (61.6)	7,796 (63.5)	11,423 (63.8)	23,987 (69.0)	29,188 (66.4)	38,161 (63.4)	40,855 (63.0)	42,508 (62.4)	49,778 (60.8)	48,884 (60.4)	46,250 (60.8)	47,389 (60.6)	44,437 (60.1)	45,934 (58.9)
CCI, median (IQR)	3 (2, 5)	3 (1, 5)	3 (1, 5)	3 (2, 5)	3 (2, 5)	3 (2, 5)	3 (2, 5)	3 (2, 5)	3 (2, 5)	3 (2, 5)	3 (2, 5)	3 (2, 5)	3 (2, 5)	3 (2, 5)	3 (2, 5)

Data are presented as mean ± SD, number (%), otherwise indicated.

*No income data.

CCI, Charlson Comorbidity Index.

**Fig 3:**
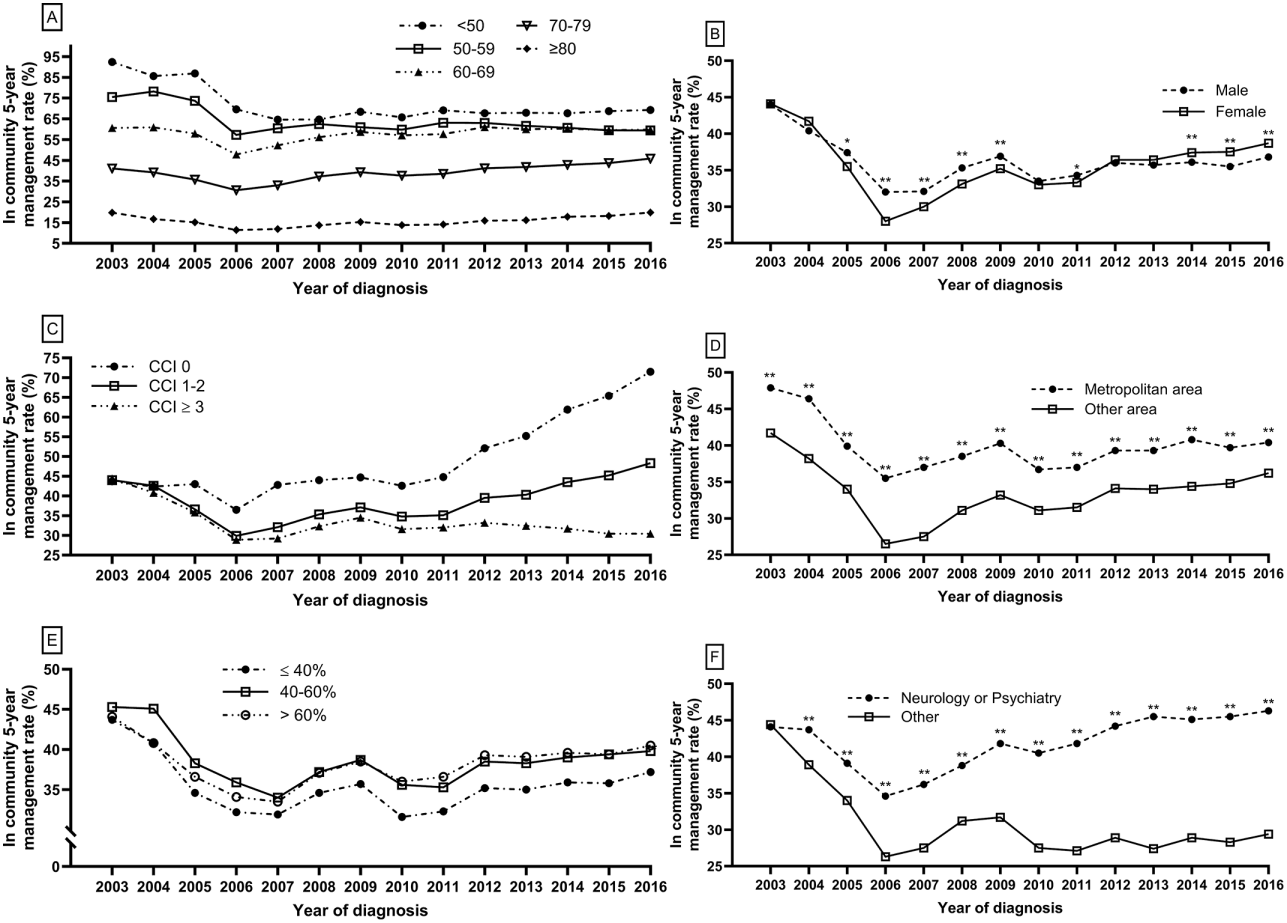
In-community 5-year management rate by subgroup. Rates were analyzed according to age **(A)**, sex **(B)**, CCI **(C)**, residence **(D)**, income **(E)**, and diagnosing department **(F)**. Metropolitan areas include Seoul, Incheon, and Gyeonggi-do. *P < 0.05, **P < 0.01 compared with the lowest group. All groups in **(A)** and all groups in **(E)** after 2004, showed significant differences compared with the lowest group.).

Participants were stratified by age, sex, CCI, area of residence, income level, and medical department at diagnosis to assess differences in the 5-year in-community management rate (Fig 3). The in-community management rate among subjects aged ≥70 years showed a gradual increase from 2014 to 2016, whereas individuals aged 50–69 exhibited a slight decrease compared to that of 2013 (Fig 3A, Supplementary figure 4). While the rate had previously been higher among males, this trend reversed in 2014, with females subsequently exhibiting a significantly higher 5-year in-community management rate than that of males (Fig 3B). From 2014 to 2016, the 5-year in-community management rate further increased among patients with no comorbidities (CCI 0), whereas rates remained relatively stable in those with moderate comorbidities (CCI 1–2) and consistently low in patients with high comorbidity burden (CCI ≥ 3, Fig 3C) Furthermore, the gap in in-community management rates between metropolitan and non-metropolitan residents gradually narrowed over time (40.8% vs 34.4% in 2014, 39.7% vs 34.8% in 2015, and 40.4% vs 36.2% in 2016; absolute differences of 6.4%, 4.9%, and 4.2%, respectively, Fig 3D). Although a significant difference was observed in the 5-year in-community management rate by income level, the gap between the lowest income group and other groups narrowed. While the rate among individuals with income levels above 40% increased by 1.4% (from 38.9% in 2013 to 40.3% in 2016), the rate in the low-income group (<40%) showed a greater increase by 2.2% (from 35.0% in 2013 to 37.2% in 2016; Fig 3E). Regarding the medical department at diagnosis, no notable difference was determined in the in-community management rate between patients diagnosed in neurology/psychiatry and those diagnosed in other departments in 2003; however, a significant divergence emerged from 2004 onward, gradually widening to a difference of over 10 percentage points in later years (Fig 3F).

## Discussion

This study presents extended data on the status of community-based management for dementia, which may serve as a key outcome indicator for evaluating South Korea’s national dementia policy. Extended data on the 5-year in-community management rate from 2014 to 2016 revealed a narrowing gap between metropolitan and non-metropolitan areas, reversal in sex-based differences, and reduction in disparities across income groups. Additionally, the newly analyzed variable, medical department at dementia diagnosis, demonstrated that patients diagnosed in neurology or psychiatry had a markedly higher in-community management rate than those diagnosed in other departments. From 2014 to 2016, the number of patients newly diagnosed with dementia remained comparable to that reported in earlier periods. The first NDP (2008–2014), referred to as the “Comprehensive Plan for Dementia Management,” was guided by the principles of promoting dignified and comfortable lives for older adults with cognitive impairment.[4] Its strategic objectives included integrating dementia care with general health promotion programs, developing tailored management approaches according to dementia subtypes, and establishing a comprehensive and systematic care infrastructure.[4] This foundational initiative, together with the enactment of the Dementia Management Act in August 2011 and its implementation in February 2012, contributed to the stabilization of early detection and treatment support programs at the national level, ultimately reinforcing the long-term effectiveness of South Korea’s structured approach to dementia care.[9] The ITS analysis also showed an increase in community management rates after the first NDP and the implementation of the Dementia Management Act in 2012, suggesting a possible causal link between these policy interventions and the observed improvements. Additionally, the 5-year in-community management rate from 2014 to 2016 showed a slight improvement compared to that of the periods before 2013. The sustained increase in this rate since 2012 may reflect the proactive management of patients with dementia through dementia support centers established as part of the National Responsibility Policy for Dementia Care announced in 2017.[5]

The World Health Organization has underscored the need for policies that facilitate early action in dementia and reduce modifiable risk factors.[2] In line with this guidance, many countries have introduced national strategies to promote early action on dementia.[10] Several policies also address care delivery and post-diagnosis support to sustain patients and caregivers.[10] Moreover, a recent multinational analysis reported lower post-diagnosis mortality after implementation of national dementia policies compared with pre-implementation periods, suggesting population-level benefits associated with these initiatives.[11] Taken together, these international data support the interpretation that iterative, policy-driven improvements—particularly those that promote earlier detection and structured community supports—can contribute to better community-based care and may help explain Korea’s favorable trajectory in 5-year in-community management rates.

There was a temporal trend toward a narrowing divide in in-community management rates between metropolitan and non-metropolitan regions from 2014 to 2016. The metropolitan areas of Korea, including Seoul, Incheon, and Gyeonggi provinces, account for approximately 51% of national population. Substantial disparities in the availability of medical facilities and number of physicians have historically existed between metropolitan and non-metropolitan areas, with little change observed around 2010.[12] However, the observed narrowing of the 5-year in-community management rate between urban and non-urban areas in 2014 and 2016 may be attributed to the expansion of dementia support centers and implementation of structured community-based dementia management strategies under national dementia policies, including the National Responsibility Policy for Dementia Care.[5,13] In particular, the Third National Dementia Plan (2016–2020) placed a strong emphasis on community-based prevention and care services, aiming to build a society where patients with dementia and their families can live comfortably and safely within their local communities.[13]

This study demonstrated an improved 5-year in-community management rate among female patients, exceeding that of their male counterparts. Women generally have a longer life expectancy than men, which often results in a higher likelihood of living alone without a spouse in later life.[14] This demographic situation may increase the risk of social isolation and physical inactivity.[15] National dementia policies, including the expansion of treatment support and introduction of the Grade 5 long-term care classification, have incorporated cognitive activity programs and home-visiting nursing services.[16] These measures likely contributed to better support for older women living alone, thereby improving their in-community management outcomes. Beyond policy-driven improvements, the gradual reduction in long‑standing gender disparities in South Korea may also have contributed to the diminishing gap. As societal attitudes toward gender equality have improved over time, access to medical evaluation and continuity of dementia care may have become more equitable between men and women.

Regarding income level, participants in the lowest 40% income group exhibited a progressive increase in their in-community management rate, leading to a reduced disparity compared to that of participants in the higher income groups. This narrowing gap may also be attributed to the implementation and refinement of the national dementia policies. Financial support for dementia-related medical expenses was first introduced under the First National Dementia Plan and was further expanded and systematized in the Second Plan.[4,17] Furthermore, the National Responsibility Policy for Dementia Care reduced the coinsurance rate for severe dementia to alleviate the financial burden on patients and their families.^5^ Programs that enabled family members of patients with dementia to register as certified caregivers also helped improve access to quality dementia care, particularly for low-income households. Low-income countries are often burdened with disproportionately high indirect costs associated with dementia care, primarily derived from unpaid caregiving responsibilities.[18] This pattern of disproportionate indirect caregiving costs observed in low-income countries may also reflect the challenges faced by low-income individuals. Measures introduced in the Third National Dementia Plan, such as the long-term care family leave program, are also expected to contribute positively by supporting the continued community-based management among economically disadvantaged individuals with dementia.[13]

Participants diagnosed and followed up by neurology or psychiatry specialists exhibited higher 5-year in-community management rates compared to those diagnosed and managed by non-dementia specialists. Patients diagnosed with dementia by non-dementia specialists may have been more likely to present with advanced disease stages or concomitant comorbid conditions requiring care in other departments. Such factors could contribute to their lower 5-year community management rates rather than reflecting differences in care quality alone. Additionally, non-dementia specialists are more likely to record dementia diagnoses as “unspecified” rather than identifying a specific subtype, even during long-term follow-up. Identifying the subtype of dementia is essential for understanding the heterogeneous clinical course and disease prognosis, which significantly impacts both patients and their caregivers.[19] Individuals diagnosed and managed by neurologists or psychiatrists may benefit from the broader clinical expertise provided by dementia specialists, facilitating more effective community-based care.[20] Due to the inadequate supply of neurologists in regional dementia centers and geriatric hospitals, national dementia policies should include strategies to systematically train and deploy dementia specialists, thereby enabling individuals with dementia to remain in their communities and live in supportive environments with caregivers.[21] These findings highlight that specialist-led dementia care are instrumental in supporting effective community management.

Meanwhile, the community 5-year management rate declined steadily from 2003 to 2006, reaching a notable low in 2006. Although no major national dementia care policy was implemented at that time, two contextual factors may have contributed to this decline. First, Korea experienced a rapid expansion in geriatric hospital beds between 2003 and 2007, which likely shifted dementia care from the community to institutional settings.[22] Second, 2006 marked the introduction of NHIS reimbursement criteria for acetylcholinesterase inhibitors, potentially bringing more advanced patients with dementia to medical attention—previously unrecognized by the medical system—to receive formal diagnoses.[23] These system-level changes may have altered the case mix and care trajectories, particularly for patients diagnosed in 2005–2006.

Although the present study provides observational evidence indicating improvements in dementia care outcomes following the implementation of national dementia policies as well as potential benefits associated with diagnosis and follow-up by neurology or psychiatry specialists, the inherent limitations of claims-based data preclude definitive conclusions regarding the optimal care pathway or specific contribution of specialist involvement. Reliance on claims data limits access to key clinical information—such as cognitive assessments (clinical dementia rating), behavioral symptoms, and neuroimaging—thereby hindering accurate classification of dementia subtypes. Although dementia subtype information can be inferred from ICD codes, our datasets consists of a customized sample that has already been filtered by dementia diagnostic criteria. This limits the ability to retrospectively reclassify cases. In addition, changes in the ICD code structure (such as the shift from 3-digit to 4-digit codes around 2010) further compromise consistency across the study period. As a result, valid subgroup analyses based on dementia subtype could not be performed. Further studies using datasets with richer clinical detail—such as cognitive test scores, structured dementia severity ratings, and validated subtype classification—are needed to assess whether community management outcomes vary by dementia subtype or severity. Moreover, the operational definition of community management cessation—defined as hospitalization or institutionalization lasting more than 3 months—was adopted based on expert consensus and prior research.[7] However, this threshold may not fully capture the complexity of patients’ functional status or caregiving dynamics. Future studies should consider evaluating alternative durations (e.g., 6 or 12 months) to validate or refine this metric. Although the setting of care (inpatient vs. outpatient) and cognitive status at initial diagnosis may have significantly influenced the community management rate, the limitations of the claims data precluded a comprehensive evaluation of these factors, underscoring the need for future research to address them for more accurate and interpretable assessments.

Second, although this study observed changes in community management rates in parallel with the implementation of national dementia policies, we could not directly evaluate the causal impact of specific policy components. Although ITS allows for evaluating policy-related changes over time and strengthens the interpretation of potential causal relationships, it remains vulnerable to confounding by concurrent external factors. The analysis cannot fully isolate the effects of specific policy components, and the absence of a control group limits the ability to rule out alternative explanations for the observed trends. Third, the analysis did not account for important individual- or community-level variables that may affect dementia care in the community, such as caregiver availability, educational level, or individual health literacy. These factors, which are not readily available in claims data, influence long-term outcomes and could introduce unobserved heterogeneity into community management patterns.

Despite these limitations, this study contributes valuable insights into the evolving management of dementia by continuously monitoring the in-community management rates. By utilizing this rate as a proxy outcome indicator, NDPs can be evaluated more objectively than by relying solely on performance-based metrics. The ongoing assessment of in-community management rates may facilitate data-driven refinement and innovation of the government-led NDPs. Furthermore, future research incorporating additional patient- and caregiver-level characteristics may broaden the scope of understanding and support more effective strategies for community-based dementia care.

## Supporting information

S1 Fig This is the S1 Fig Title. (PDF)

S2 Fig This is the S2 Fig Title. (PDF)

S3 Fig This is the S3 Fig Title. (PDF)

S4 Fig This is the S4 Fig Title. (PDF)

S1 Table This is the S1 Table Title. (PDF)

S2 Table This is the S2 Table Title. (PDF)
