# Supplementary material for: Trends in 5-year community management of persons with dementia in Korea, 2003–2016
Source: PLoS One. 2026 Mar 11;21(3):e0342459. doi: 10.1371/journal.pone.0342459 (PMC12978433; doi:10.1371/journal.pone.0342459)
Supplement: S2 Fig — (PDF) [file pone.0342459.s002.pdf]

Supplementary figure 2. Age-standardized 5-year community management rates

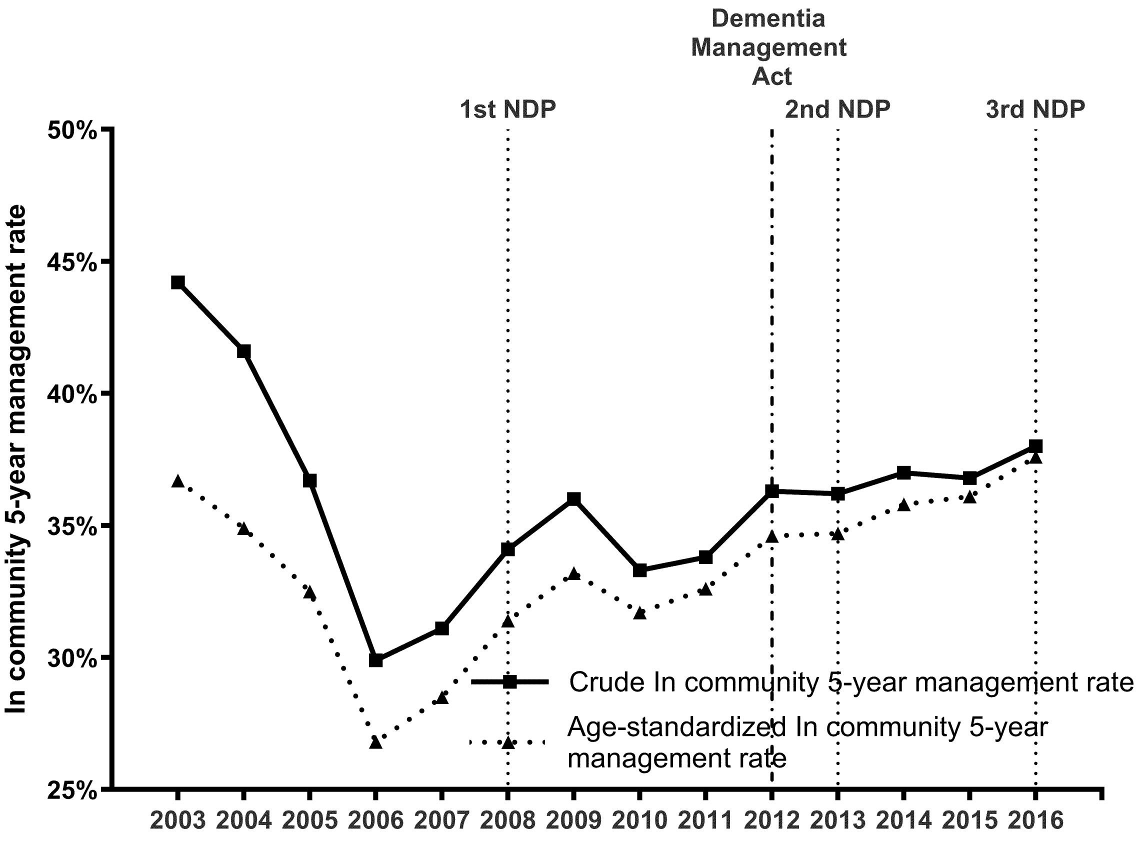

NDP: National dementia policy
