# Supplementary material for: Trends in 5-year community management of persons with dementia in Korea, 2003–2016
Source: PLoS One. 2026 Mar 11;21(3):e0342459. doi: 10.1371/journal.pone.0342459 (PMC12978433; doi:10.1371/journal.pone.0342459)
Supplement: S4 Fig — (PDF) [file pone.0342459.s004.pdf]

Supplementary figure 4. The sample size (n) for each subgroup as classified by age

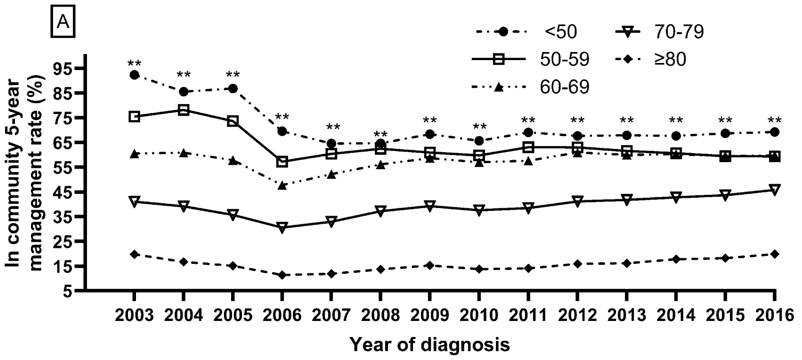

	Year	2003	2004	2005	2006	2007	2008	2009	2010	2011	2012	2013	2014	2015	2016
Age															
- 49	Total subjects with dementia	237	270	282	674	840	1,150	1,303	1,232	1,504	1,671	1,500	1,587	1,449	1,564
	Patients remaining under community management for five years	219	231	245	469	543	744	891	809	1,040	1,132	1,018	1,074	995	1,084
50-59	Total subjects with dementia	503	706	877	1,888	2,272	3,269	3,681	3,581	4,295	4,591	4,370	4,413	4,020	4,420
	Patients remaining under community management for five years	380	552	646	1,081	1,373	2,042	2,247	2,140	2,710	2,893	2,691	2,678	2,391	2,625
60-69	Total subjects with dementia	2,045	2,779	3,549	6,228	7,582	10,308	10,955	10,610	11,906	11,700	10,797	10,996	10,433	11,008
	Patients remaining under community management for five years	1,240	1,693	2,056	2,975	3,959	5,795	6,434	6,051	6,874	7,137	6,475	6,635	6,217	6,579
70-79	Total subjects with dementia	3,390	5,028	7,277	13,646	16,965	23,215	25,405	26,250	31,980	32,000	29,711	30,119	27,521	27,705
	Patients remaining under community management for five years	1,393	1,972	2,597	4,182	5,592	8,647	9,991	9,869	12,328	13,176	12,412	12,903	12,016	12,689
80 -	Total subjects with dementia	2,108	3,384	5,722	11,923	15,760	21,771	23,133	25,985	31,716	30,699	29,398	30,791	30,439	33,196
	Patients remaining under community management for five years	417	566	862	1,365	1,880	2,980	3,528	3,581	4,460	4,866	4,771	5,477	5,540	6,599
