# Supplementary material for: Trends in 5-year community management of persons with dementia in Korea, 2003–2016
Source: PLoS One. 2026 Mar 11;21(3):e0342459. doi: 10.1371/journal.pone.0342459 (PMC12978433; doi:10.1371/journal.pone.0342459)
Supplement: S1 Table — (PDF) [file pone.0342459.s005.pdf]

Supplementary table 1. Diagnostic categories, weights, and corresponding ICD-10 codes of Charlson comorbidity index

Condition	Weights	ICD-10 codes
Acute myocardial infarction	1	I21, I22, I252
Congestive heart failure	1	I50
Peripheral vascular disease	1	I71, I790, I739, R02, Z958, Z959
Cerebral vascular accident	1	I60, I61, I62, I63, I65, I66,G450, G451, G452, G458, G459, G46, I64, G454, I670, I671, I672, I674, I675, I676, I677 I678, I679, I681, I682, I688, I69
Dementia	1	F00, F01, F02, F051
Pulmonary disease	1	J40, J41, J42, J44, J43, J45, J46, J47, J67, J44, J60, J61, J62, J63, J66, J64, J65
Connective tissue disorder	1	M32, M34, M332, M053, M058, M059, M060, M063, M069, M050, M052, M051, M353
Peptic ulcer	1	K25, K26, K27, K28
Mild liver disease	1	K702, K703, K73, K717, K740, K742, K746, K743, K744, K745
Diabetes	1	E109, E119, E139, E149, E101, E111, E131, E141, E105, E115, E135, E145
Diabetes with complications	2	E102, E112, E132, E142 E103, E113, E133, E143 E104, E114, E134, E144
Paraplegia	2	G81 G041, G820, G821, G822
Renal disease	2	N03, N052, N053, N054, N055, N056, N072, N073, N074, N01, N18, N19, N25

Cancer	2	C0, C1, C2, C3, C40, C41, C43, C45, C46, C47, C48, C49, C5, C6, C70, C71, C72, C73, C74, C75, C76, C80, C81, C82, C83, C84, C85, C883, C887, C889, C900, C901, C91, C92, C93, C940, C941, C942, C943, C9451, C947, C95, C96
Metastatic cancer	3	C77, C78, C79, C80
Severe liver disease	3	K729, K766, K767, K721
HIV	6	B20, B21, B22, B23, B24
