# Supplementary material for: Trends in 5-year community management of persons with dementia in Korea, 2003–2016
Source: PLoS One. 2026 Mar 11;21(3):e0342459. doi: 10.1371/journal.pone.0342459 (PMC12978433; doi:10.1371/journal.pone.0342459)
Supplement: S2 Table — (PDF) [file pone.0342459.s006.pdf]

Supplementary table 2. Subgroup-Specific 5-Year Community Management Rates Shown in Figure 3

A. age (%)

		2003	2004	2005	2006	2007	2008	2009	2010	2011	2012	2013	2014	2015	2016
<50	5-year management rate	(92.4)	(85.6)	(86.9)	(69.6)	(64.6)	(64.7)	(68.4)	(65.7)	(69.1)	(67.7)	(67.9)	(67.7)	(68.7)	(69.3)
50-59	5-year management rate	(75.5)	(78.2)	(73.7)	(57.3)	(60.4)	(62.5)	(61.0)	(59.8)	(63.1)	(63.0)	(61.6)	(60.7)	(59.5)	(59.4)
60-69	5-year management rate	(60.6)	(60.9)	(57.9)	(47.8)	(52.2)	(56.2)	(58.7)	(57.0)	(57.7)	(61.0)	(60.0)	(60.3)	(59.6)	(59.8)
70-79	5-year management rate	(41.1)	(39.2)	(35.7)	(30.6)	(33.0)	(37.2)	(39.3)	(37.6)	(38.5)	(41.2)	(41.8)	(42.8)	(43.7)	(45.8)
>80	5-year management rate	(19.8)	(16.7)	(15.1)	(11.4)	(11.9)	(13.7)	(15.3)	(13.8)	(14.1)	(15.9)	(16.2)	(17.8)	(18.2)	(19.9)

B. sex (%)

		2003	2004	2005	2006	2007	2008	2009	2010	2011	2012	2013	2014	2015	2016
Male	5-year management rate	(44.0)	(40.4)	(37.4)	(32.0)	(32.1)	(35.3)	(36.9)	(33.5)	(34.3)	(36.0)	(35.7)	(36.1)	(35.5)	(36.8)
Female	5-year management rate	(44.1)	(41.7)	(35.5)	(28.0)	(30.1)	(33.1)	(35.2)	(33.0)	(33.4)	(36.3)	(36.4)	(37.4)	(37.5)	(38.7)

C. CCI (%)

		2003	2004	2005	2006	2007	2008	2009	2010	2011	2012	2013	2014	2015	2016
0	5-year management rate	(43.7)	(42.4)	(43.0)	(36.5)	(42.8)	(44.0)	(44.7)	(42.6)	(44.8)	(52.1)	(55.2)	(61.9)	(65.4)	(71.5)
1-2	5-year management rate	(44.0)	(42.6)	(36.6)	(29.9)	(32.1)	(35.3)	(37.1)	(34.8)	(35.1)	(39.5)	(40.3)	(43.5)	(45.2)	(48.3)
>3	5-year management rate	(44.4)	(40.8)	(35.8)	(28.9)	(29.2)	(32.3)	(34.5)	(31.6)	(32.0)	(33.2)	(32.4)	(31.7)	(30.4)	(30.4)

D. residence (%)

		2003	2004	2005	2006	2007	2008	2009	2010	2011	2012	2013	2014	2015	2016
Metropolitan	5-year management rate	(47.9)	(46.4)	(39.9)	(35.5)	(37.0)	(38.5)	(40.3)	(36.7)	(37.0)	(39.3)	(39.3)	(40.8)	(39.7)	(40.4)
Non-metropolitan	5-year management rate	(41.7)	(38.2)	(34.0)	(26.5)	(27.5)	(31.1)	(33.2)	(31.1)	(31.5)	(34.1)	(34.0)	(34.4)	(34.8)	(36.2)

E. income (%)

		2003	2004	2005	2006	2007	2008	2009	2010	2011	2012	2013	2014	2015	2016
<40%	5-year management rate	(43.7)	(40.8)	(34.6)	(32.2)	(31.9)	(34.6)	(35.7)	(31.6)	(32.3)	(35.2)	(35.0)	(35.9)	(35.8)	(37.2)
40-60%	5-year management rate	(45.3)	(45.1)	(38.3)	(35.9)	(34.0)	(37.2)	(38.7)	(35.6)	(35.3)	(38.5)	(38.3)	(39.0)	(39.4)	(39.8)
>60%	5-year management rate	(44.1)	(40.8)	(36.6)	(34.1)	(33.5)	(37.1)	(38.5)	(36.0)	(36.6)	(39.3)	(39.1)	(39.6)	(39.4)	(40.5)

F. diagnosing department (%)

		2003	2004	2005	2006	2007	2008	2009	2010	2011	2012	2013	2014	2015	2016
Neurology or Psychiatry	5-year management rate	(44.1)	(43.7)	(39.1)	(34.6)	(36.2)	(38.8)	(41.8)	(40.5)	(41.8)	(44.2)	(45.5)	(45.1)	(45.5)	(46.3)
Others	5-year management rate	(44.4)	(38.9)	(34.0)	(26.3)	(27.5)	(31.2)	(31.7)	(27.5)	(27.1)	(28.9)	(27.4)	(28.9)	(28.3)	(29.4)

CCI: Charlson comorbidity index
